# Supplementary material for: Protection of the C. elegans germ cell genome depends on diverse DNA repair pathways during normal proliferation
Source: PLoS One. 2021 Apr 27;16(4):e0250291. doi: 10.1371/journal.pone.0250291 (PMC8078821; doi:10.1371/journal.pone.0250291)
Supplement: S1 File — (DOCX) [file pone.0250291.s017.docx]

**Meier et al., 2020: Protection of the *C. elegans* germ cell genome depends on diverse DNA repair pathways during normal proliferation**

**S1 File.** Filtered variant calling VCF files for 31 samples that have not been made publicly available previously [1] and are unique to this study.

**Supplementary References**

1. [Volkova NV, Meier B, González-Huici V, Bertolini S, Gonzalez S, Vöhringer H, et al. Mutational signatures are jointly shaped by DNA damage and repair. Nat Commun. 2020;11: 2169.](http://paperpile.com/b/jV5yVq/l8bkN)
